# Embracing the taxonomic and topological stability of phylogenomics

**DOI:** 10.1038/s41598-024-54208-4

**Published:** 2024-02-19

**Authors:** Nicolás Mongiardino Koch

**Affiliations:** Scripps Institution of Oceanography, University of California San Diego, La Jolla, CA USA

**Keywords:** Phylogenetics, Molecular evolution, Taxonomy

**Arising from**: H. Lee et al.; *Scientific Reports* 10.1038/s41598-023-36848-0 (2023).

The classification of sand dollars was recently reassessed by Lee et al*.*^1^ based on a four-locus molecular dataset. While expanding the taxon sampling relative to previous studies, and providing a novel hypothesis of relationship for some sand dollar lineages, the authors also favor a topology that incorporates several deep splits that are incongruent with previous morphological and molecular efforts, including several genome-scale studies. Here, I reevaluate their dataset and find that it does not harbor the necessary signal to resolve deep branching patterns. On the contrary, available phylogenomic data reject their tree as a plausible phylogenetic hypothesis. Conflicting phylogenetic trees should not be considered for taxonomic or macroevolutionary purposes without first evaluating the adequacy of the data at hand, especially when publicly available, genome-scale datasets for the intervening taxa already exist.

Phylogenetic trees serve two major purposes: (1) they provide a means for classifying organisms into taxonomic groups, and (2) they are the substrate on which evolutionary patterns and processes are explored. In a recent publication, Lee et al*.*^1^ presented a novel hypothesis of relationships for sand dollars, sea biscuits, and close relatives, a clade of echinoids known as Luminacea. Their phylogenetic tree was then used as mentioned above to introduce a new classification of sand dollars, suggest potential morphological homoplasies, and infer the biogeographic history of the clade. The efforts of Lee et al*.*^1^, however, support a topology that is at odds with our current understanding of the phylogeny of irregular echinoids, while disregarding the wealth of molecular resources already available for the clade^2^.

As noted by the authors, the classification of luminacean lineages has been heavily revised recently, suggesting a much more dynamic morphological evolution than once considered^3,4^ (Fig. 1A–C). In fact, the name Luminacea was only proposed two years ago. Nonetheless, these taxonomic changes were supported by genome-scale datasets^2,5^, congruent with previous small-scale molecular studies^6^, and adopted only after a thorough assessment of levels of phylogenetic signal, noise, and biases in the data. Lee et al.^1^ gathered a novel molecular dataset for Luminacea composed of four loci (*16 s*, *18 s*, *cox1*, and *H3*) and including the broadest sample of sand dollar (Scutelloida) family-level clades. Their efforts help clarify relationships among Scutelliformes, supporting the erection of three new superfamilies, and posing valuable hypotheses regarding the independent origins of lunules (the unique perforations in the tests of some sand dollars). Nonetheless, their topology (shown in Fig. 1C) markedly departs from those of recent studies (shown in Fig. 1B), rejecting the monophyly of sand dollars and finding a novel placement for Cassiduloida. This tree has no precedent in the literature, conflicting with Sanger-sequenced^6^, mitogenomic^7^, transcriptomic^2,5^, and total-evidence^8^ datasets, and further widening the discrepancy between molecular and morphological evidence (see Figs. 1 and S1; increased morphological tree lengths reflect elevated homoplasy). Despite these challenges, Lee et al*.*^1^ embrace their novel results, citing the benefits of a wider taxon sampling, and proposing that morphological similarities among sand dollars arose through convergent evolution.

Luminacea is a relatively ancient clade of irregular echinoids that originated sometime between the Middle Jurassic^8^ and the Early Cretaceous^1,2^, and which displays elevated heterogeneity in evolutionary rates^5^. These conditions pose substantial obstacles for phylogenetic reconstruction, especially when relying on small-scale datasets. In line with this, deep nodes in the phylogeny of Lee et al.^1^ show low support values, including those relating to the novel rearrangements proposed. In order to assess the robustness of their results, I obtained the data used by the authors from GenBank (72 nucleotide sequences for 28 taxa, encompassing four loci with occupancies ranging between 25 and 96%). Given the lack of *H3* sequences among outgroup taxa, sequences of *Strongylocentrotus purpuratus* were further incorporated. Loci were aligned using the L-INS-i algorithm in MAFFT v7.505^9^, and concatenated into a matrix both with and without sequences for *S. purpuratus* (the latter replicating the original dataset, although the authors manually aligned some loci). The effect of trimming positions with over 50% missing data was also explored, determining four concatenated matrices. Inference from these datasets, as well as others mentioned below, was performed under maximum likelihood using optimal partitioned models in IQ-TREE v1.6.12^10,11^. Despite differing minimally with respect to the original alignment, none of these datasets recovered the topology supported by Lee et al.^1^ (Fig. S2), an instability that could indicate low levels of phylogenetic signal.

To assess this hypothesis, and establish whether the dataset gathered by Lee et al.^1^ is able to confidently resolve relationships among the major lineages within Luminacea (i.e., Cassiduloida, Clypeasteroida, Laganiformes, and Scutelliformes; see Fig. 1) I relied on approximately unbiased (AU) topological tests^12^. I performed a set of constrained tree searches enforcing the monophyly of the four aforementioned clades as well as of the nodes connecting them, exploring all fifteen possible patterns of relationships among them. All other nodes were left unconstrained. Comparison of the likelihood scores of these candidate trees reveals that five topological alternatives, including those shown in Fig. 1B and C, form part of the confidence set of trees (Table S1). These results prove that the data of Lee et al*.*^1^ cannot be used to support (or reject) deep relationships within Luminacea, and that their results do not truly deviate from those of previous studies.

While phylogenomic datasets sometimes fail to uncover true phylogenetic histories, the amount of information they contain can be much more thoroughly explored, allowing for competing signals to be quantified and diagnosed. I reanalyzed the latest echinoid phylotranscriptomic dataset^2^, estimating site-wise log likelihood scores for the trees depicted in Fig. 1B and C. These were turned into gene-wise scores whose difference, known as ΔGLS^13^, represent amounts of signal for/against topological alternatives. As shown in Fig. 1D, phylogenomic data strongly support the monophyly of Scutelloida, as well as the placement of Cassiduloida as their sister clade. On the other hand, the tree put forth by Lee et al.^1^ finds only minimal support, being the preferred topology for only 8% of loci.

**Figure 1:**
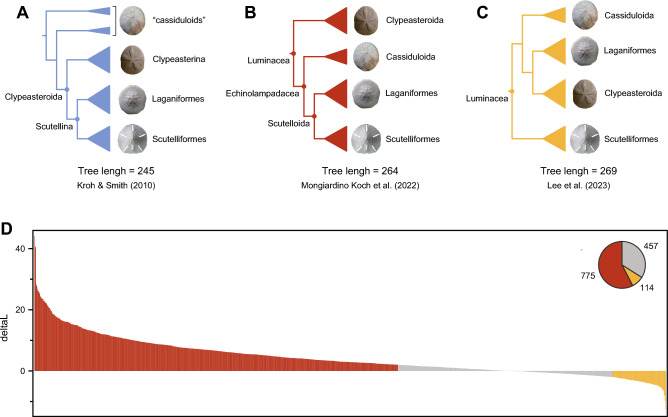
Topology, classification, and phylogenetic signal for relationships between sand dollars, sea biscuits, and close relatives. (**A**) Traditional morphological hypothesis. (**B**) Phylogenomic topology and classification^2,5^. (**C**) Phylogeny recently proposed by Lee et al*.*^1^. Clade width is scaled to extant diversity, compiled from the World Echinoidea Database^13^. “Cassiduloids” and Cassiduloida refer here to the families Cassidulidae and Echinolampadidae. Tree length is the maximum parsimony score of the three topologies using the morphological dataset of Kroh & Smith^3^, estimated with TNT v1.5^14^ using topological constraints. Larger scores reflect increased conflicts between molecular and morphological evidence. Inferred morphological trees can be found in Fig. S1. (**D**) Distribution of delta gene-wise log-likelihood scores (ΔGLS) across the phylogenomic dataset of Mongiardino Koch et al*.*^2^. Topologies tested are those shown in Fig. 1B and C, and the same colors are used to depict loci favoring each. Uninformative genes, defined as those with absolute ΔGLS < 2, are shown in grey.

Our understanding of the phylogeny and evolutionary history of sand dollars, sea biscuits, and close relatives, has changed dramatically in recent years^2,5,8^, prompting a taxonomic restructuring. Change, however, does not necessarily reflect uncertainty. The available molecular data for Luminacea places us, for the first time, in a position in which we can build a stable taxonomic classification for its living members. Improved taxon sampling will continue to provide novel phylogenetic insights; however, favoring phylogenies that conflict with those built using thousands of loci amounts to an extraordinary claim, one that, as shown here, is not based on sufficient evidence. While the phylogenetic and evolutionary hypotheses put forth by the authors for scutelliforms are valuable, their data do not substantiate a phylogenetic reassessment of Luminacea. Doing so threatens to perpetuate a state of taxonomic instability that is unwarranted in light of the data at hand, as well as potentially lead to inaccurate inferences of morphological, biogeographical, and macroevolutionary history.

## Additional information

Supplementary information for this paper, including figures and tables, is available online. Data, results, and code to replicate all aspects of the analysis is deposited in the Zenodo repository: 10.5281/zenodo.10207759.

### Supplementary Information

Supplementary Information.
