# Supplementary material for: Embracing the taxonomic and topological stability of phylogenomics
Source: Sci Rep. 2024 Feb 19;14:4088. doi: 10.1038/s41598-024-54208-4 (PMC10876614; doi:10.1038/s41598-024-54208-4)
Supplement: Supplementary file 1 — Supplementary Information. [file 41598_2024_54208_MOESM1_ESM.docx]

**Supplementary Material – Embracing the taxonomic and topological stability of phylogenomics**

Nicolás Mongiardino Koch


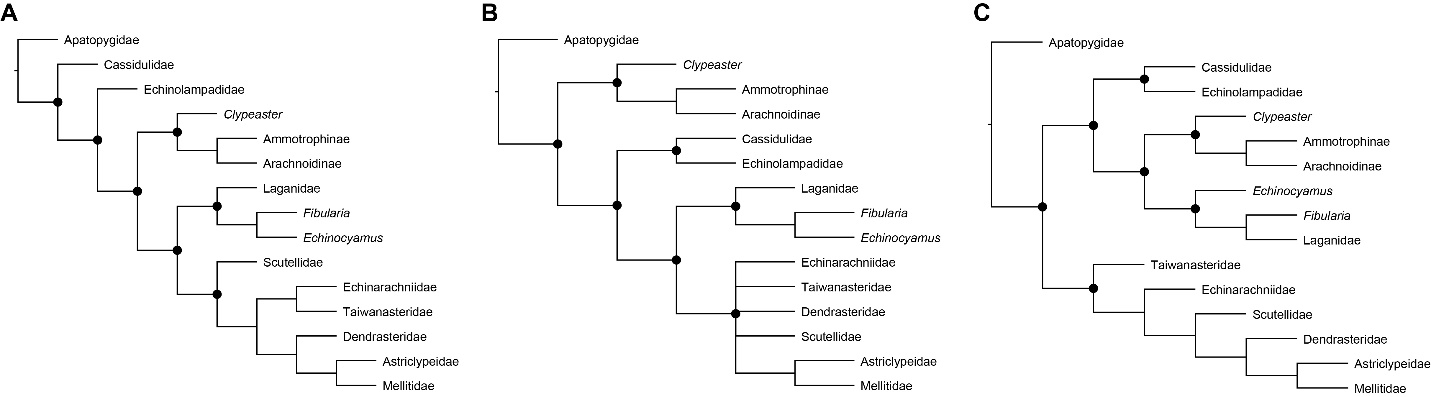


**Figure S1:** Morphological trees replicating the results of **A)** Kroh & Smith (2010), a morphological analysis; **B)** Mongiardino Koch *et al.* (2020), a phylotranscriptomic analysis; and **C)** a Lee *et al.* (2023), a study based on Sanger-sequenced data. Each analysis was run in TNT v1.5 (Goloboff & Catalano 2015), using the morphological dataset of Kroh & Smith (2010) and constraining 7 nodes which are marked with black circles. Topologies were rooted with Apatopygidae, the sister-group to Luminacea. Topologies **B** and **C** are strict consensuses of 3 and 6 most-parsimonious trees, respectively. Fossil terminals and clades not sampled in molecular analyses (Rotulidae and Neolampadidae) were removed before inference, along with all other outgroup taxa. As reported in Figure 1, tree lengths are 245, 264, and 269, respectively.


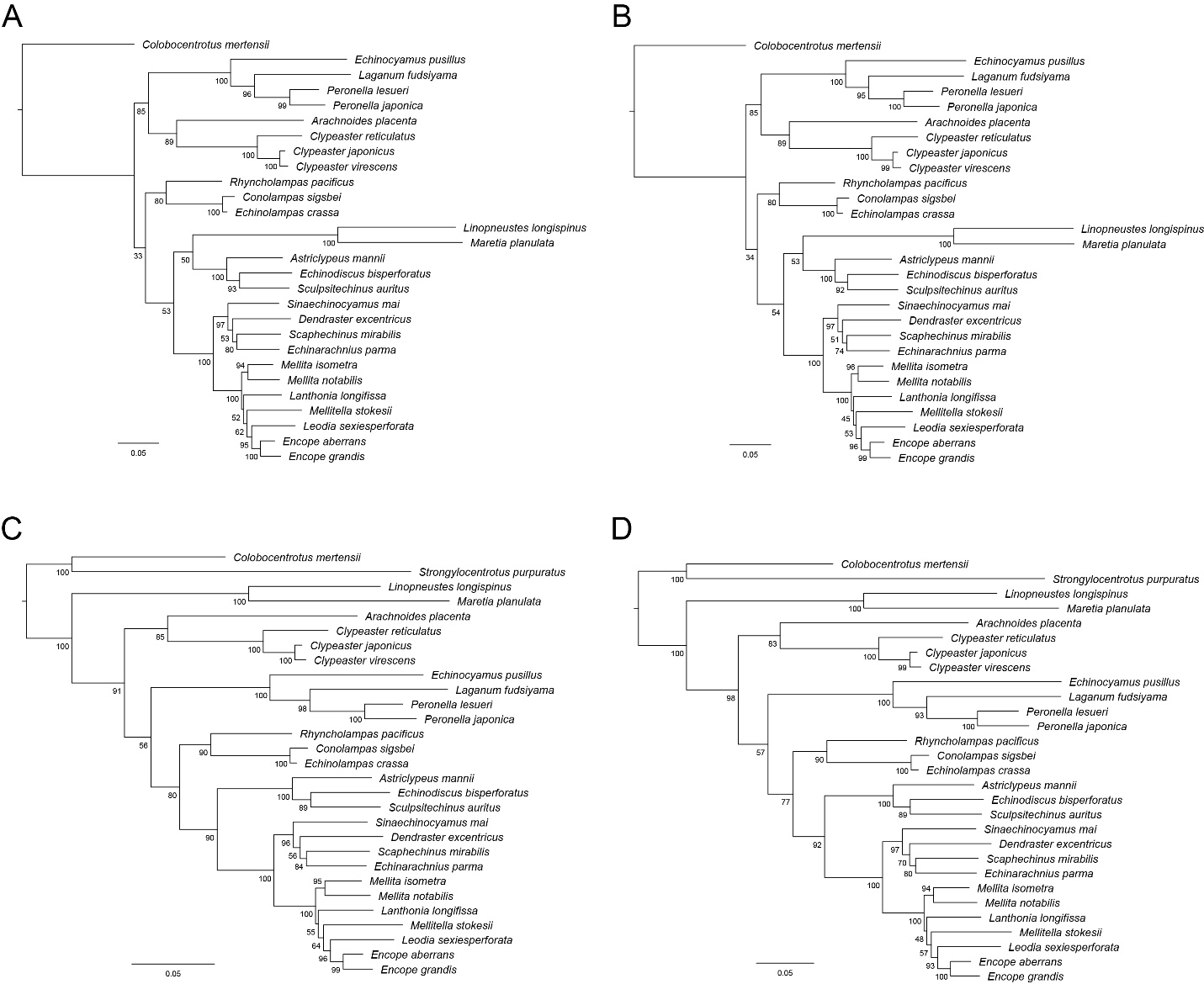


**Figure S2:** Optimal topologies inferred under partitioned ML for the four concatenated supermatrices analyzed. **A)** Untrimmed, without *S. purpuratus*. **B)** Trimmed, without *S. purpuratus*. **C)** Untrimmed, with *S. purpuratus*. **D)** Trimmed, with *S. purpuratus*. Condition **A** replicates the matrix of Lee *et al.* (2023), yet the use of a different alignment approach, as well as different model and inference software, leads to the recovery of a different topology than that reported by the authors. Neither tree agrees with the topologies of either Lee *et al.* (2023) nor Mongiardino Koch *et al.* (2022), not even if spatangoids are pruned from their incorrect placement in **A** and **B**.

**Table S1:** Results of the AU tests of all possible topologies for major luminacean clades. Results are shown for each individual locus, as well as for the concatenated supermatrix. There are no *H3* sequences for cassiduloids in the dataset, thus the topological space is composed of only three options. All trees were rooted with at least one outgroup. The data correspond to the trimmed alignment including sequences of *S. purpuratus* (otherwise testing of *H3* is impossible). Numbers correspond to *P*-values; all topologies with *P* > 0.05 form part of the confidence set of trees, and are denoted with a “+”. The overall size of confidence sets is denoted at the bottom. The topologies being considered (i.e., those shown in Figs. 1B and 1C) are denoted. Ca = Cassiduloids, Cl = Clypeasteroida, L = Laganiformes, S = Scutelliformes.

| **Topology** | ***16s*** | ***28s*** | ***cox1*** | **supermatrix** |
| --- | --- | --- | --- | --- |
| (Ca, (L, (S, Cl))) | 0.0085 | 0.0067 | 0.0330 | 0.0000 |
| (Ca, (Cl, (L, S))) | 0.0149 | 0.0021 | **+ 0.2940** | 0.0020 |
| (Ca, (S, (Cl, L))) | **+ 0.1180** | 0.0103 | **+ 0.2310** | 0.0094 |
| (Cl, (Ca, (L, S))) – Fig. 1C | 0.0387 | **+ 0.8180** | **+ 0.7150** | **+ 0.2640** |
| (Cl, (S, (Ca, L))) | 0.0040 | **+ 0.1890** | **+ 0.5260** | 0.0387 |
| (Cl, (L, (S, Ca))) | **+ 0.1580** | **+ 0.1890** | **+ 0.5240** | **+ 0.7650** |
| (L, (S, (Ca, Cl))) | **+ 0.0692** | **+ 0.0908** | **+ 0.1110** | 0.0240 |
| (L, (Cl, (S, Ca))) | **+ 0.2360** | 0.0003 | **+ 0.0990** | 0.0077 |
| (L, (Ca, (Cl, S))) | 0.0169 | 0.0067 | **+ 0.1030** | 0.0000 |
| (S, (Ca, (L, Cl))) – Fig. 1B | **+ 0.2720** | **+ 0.1800** | **+ 0.7670** | **+ 0.3910** |
| (S, (Cl, (Ca, L))) | 0.0157 | 0.0281 | **+ 0.3350** | 0.0120 |
| (S, (L, (Cl, Ca))) | **+ 0.0694** | **+ 0.5450** | **+ 0.3370** | **+ 0.0977** |
| ((Ca, L), (S, Cl)) | 0.0094 | 0.0000 | **+ 0.0577** | 0.0000 |
| ((Ca, Cl), (L, S)) | **+ 0.0719** | **+ 0.1400** | **+ 0.4620** | 0.0332 |
| ((Ca, S), (Cl, L)) | **+ 0.9380** | 0.0059 | **+ 0.3810** | **+ 0.4990** |
| **overall:** | 8/15 (53.0%) | 7/15 (46.7%) | 14/15 (93.3%) | 5/15 (33.3%) |
|  |  |  |  |  |
| **Topology** | ***H3*** |  |  |  |
| (L, (Cl, S)) | **+ 0.1500** |  |  |  |
| (Cl, (L, S)) – Fig. 1C | **+ 0.6600** |  |  |  |
| (S, (Cl, L)) – Fig. 1B | **+ 0.4270** |  |  |  |
| **overall:** | 3/3 (100%) |  |  |  |
